# Ataxin-2-Like Is a Regulator of Stress Granules and Processing Bodies

**DOI:** 10.1371/journal.pone.0050134

**Published:** 2012-11-27

**Authors:** Christian Kaehler, Jörg Isensee, Ute Nonhoff, Markus Terrey, Tim Hucho, Hans Lehrach, Sylvia Krobitsch

**Affiliations:** 1 Otto Warburg Laboratory, Max Planck Institute for Molecular Genetics, Berlin, Germany; 2 Department of Human Molecular Genetics, Max Planck Institute for Molecular Genetics, Berlin, Germany; 3 Department of Vertebrate Genomics, Max Planck Institute for Molecular Genetics, Berlin, Germany; 4 Free University Berlin, Department of Biology, Chemistry and Pharmacy, Berlin, Germany; 5 Universitätsklinik Köln, Klinik für Anästhesiologie und Operative Intensivmedizin, Experimentelle Anästhesiologie und Schmerzforschung, Cologne, Germany; Ohio State University, United States of America

## Abstract

Paralogs for several proteins implicated in neurodegenerative disorders have been identified and explored to further facilitate the identification of molecular mechanisms contributing to disease pathogenesis. For the disease-causing protein in spinocerebellar ataxia type 2, ataxin-2, a paralog of unknown function, termed ataxin-2-like, has been described. We discovered that ataxin-2-like associates with known interaction partners of ataxin-2, the RNA helicase DDX6 and the poly(A)-binding protein, and with ataxin-2 itself. Furthermore, we found that ataxin-2-like is a component of stress granules. Interestingly, sole ataxin-2-like overexpression led to the induction of stress granules, while a reduction of stress granules was detected in case of a low ataxin-2-like level. Finally, we observed that overexpression of ataxin-2-like as well as its reduction has an impact on the presence of microscopically visible processing bodies. Thus, our results imply a functional overlap between ataxin-2-like and ataxin-2, and further indicate a role for ataxin-2-like in the regulation of stress granules and processing bodies.

## Introduction

Late-onset neurodegenerative disorders have been intensively studied over the last two decades. However, the molecular mechanisms responsible for their pathologies remain to be elucidated. Of note, some knowledge was gained by exploring the physiological function of paralogous proteins identified for several disease proteins. Regarding the family of polyglutamine disorders, which includes Huntington’s disease, spinobulbar muscular atrophy, dentatorubral pallidoluysian atrophy, and spinocerebellar ataxia (SCA) type 1, 2, 3, 6, 7 & 17 [1], [2], [3], [4], a gene duplication of ataxin-1-like (ATXN1L)/Brother of ataxin-1 (Boat), the respective paralog of the disease-causing protein ataxin-1 (ATXN1), ameliorated the observed neurotoxicity in a SCA1 mouse model, indicating overlapping functionality between paralog and disease protein [5].

The search for the gene causing SCA2 led to the isolation of the *SCA2* gene [6], [7], [8], which comprises an intrinsic CAG repeat that is interrupted by 1–3 CAA triplets in healthy individuals, while a continuous CAG repeat of more than 34 repeats has been observed in affected individuals [6], [8], [9]. The expansion on the genetic level is causal for an extended polyglutamine domain in the *SCA2* gene product ataxin-2 (ATXN2). Interestingly, these efforts also resulted in the isolation of a partial cDNA sequence on chromosome 16 that showed high homology to the *SCA2* gene sequence [7]. The encoded protein showed high homology with ATXN2 and was therefore named ataxin-2-related protein (A2RP) [10]. Independently from these studies, Meunier and colleagues reported the identification of a gene at the same chromosome locus and named the respective gene product ataxin-2 domain protein (A2D) [11]. Proteins of the A2RP or A2D family, which we refer to as ataxin-2-like (ATXN2L), are widely expressed in human tissues and orthologs are present in other species [10]. Comparison of the derived amino acid sequences of ATXN2 and ATXN2L showed that several motifs are conserved such as the N-terminal acidic domain containing the mRNA-binding motifs Sm1 and Sm2, putative caspase-3 cleavage sites, a clathrin-mediated trans-Golgi signal, and an endoplasmic reticulum exit signal. Furthermore, both proteins comprise the binding motif for the PABC domain of the poly(A)-binding protein (PABP), termed PAM2 [10], [12]. Despite these shared motifs, the polyglutamine tract is not conserved between ATXN2 and ATXN2L [10].

Considering the high degree of structural similarity between ATXN2 and ATXN2L, a functional overlap between these paralogs is likely. Regarding the cellular function of ATXN2L, which remains to be understood, an association with the erythropoietin receptor has been reported suggesting a function in cytokine signaling [11]. To this point, a role of ATXN2 in endocytic processes and RNA-processing pathways was demonstrated [13], [14], [15], [16], [17]. Concerning its function in the cellular RNA metabolism, ATXN2 is found in association with PABP, further being a dosage-dependent regulator of this protein [14], [15]. Moreover, direct interactions of ATXN2 with RNA splicing factors and RNA-binding proteins have been described [18], [19]. Finally, an association of ATXN2 with polyribosomes and direct binding of ATXN2 to RNA was demonstrated [20], and ATXN2 has been identified as a component of stress granules (SGs) [14], [15]. These are dynamic cellular structures assembling in mammalian cells in response to diverse cellular stresses representing sites of mRNA regulation. SGs contain untranslated mRNAs, eukaryotic initiation factors, small ribosomal subunits, various RNA-binding proteins, and proteins implicated in cell signaling [21], [22], [23]. Furthermore, there is a dynamic interplay between SGs and processing bodies (P-bodies), sites of mRNA degradation that comprise components of microRNA or RNAi pathways as well as the nonsense-mediated mRNA decay pathway [24].

In this study, we considered a potential functional overlap between ATXN2L and ATXN2 with regard to RNA metabolism. We discovered that ATXN2L associates with known ATXN2 interaction partners such as the RNA helicase DDX6 and PABP, and with ATXN2 itself. Furthermore, we observed that ATXN2L is a *bona fide* component of SGs. Finally, we report that ATXN2L overexpression as well as its reduction has a regulatory effect on SGs and P-bodies.

## Results

### ATXN2L Associates with PABP, DDX6, and ATXN2

**Figure 1:**
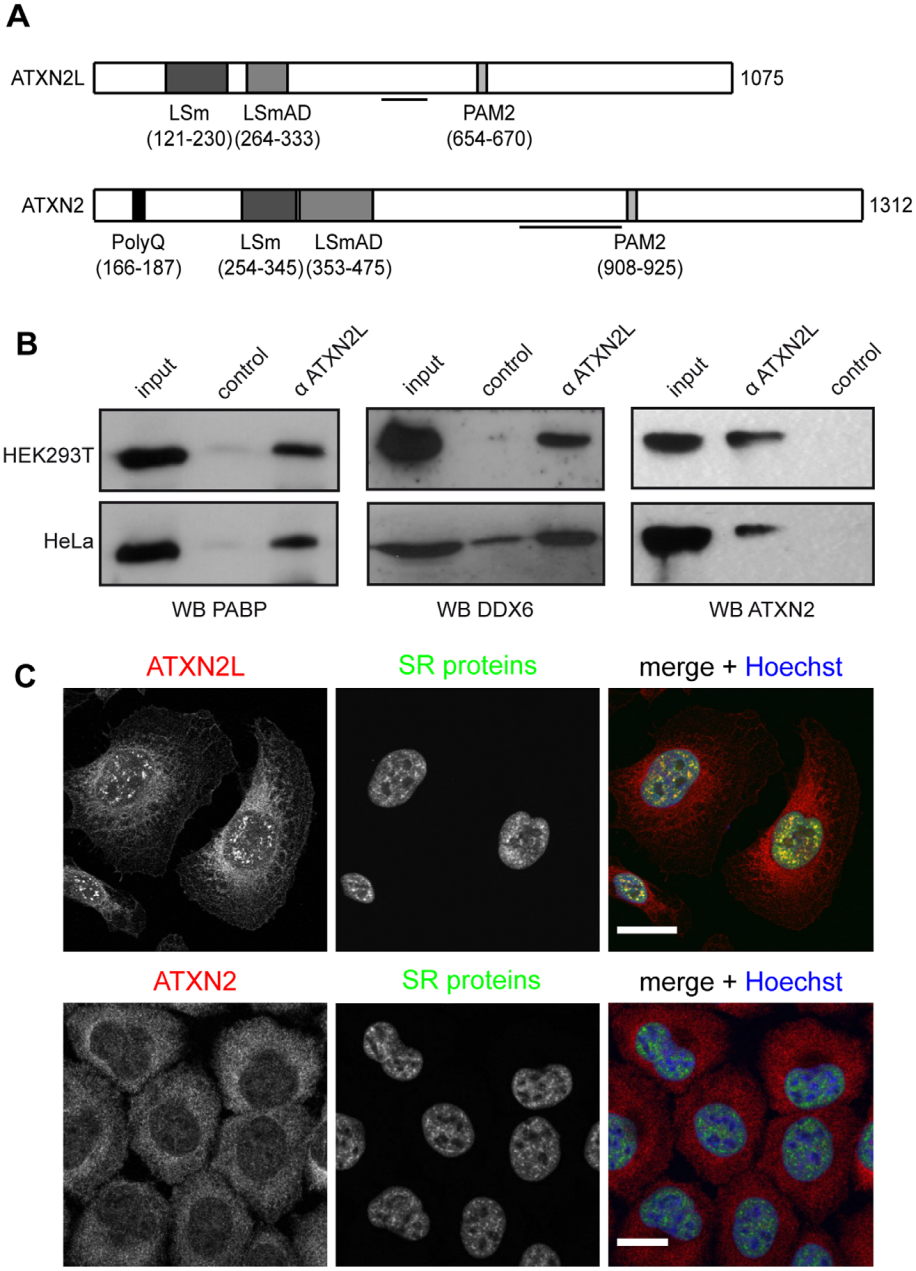
ATXN2L is found in association with PABP, DDX6 and ATXN2. A) Scheme of the domain architecture of ATXN2L and ATXN2 highlighting conserved functional motifs. Lines below indicate antibody epitopes for anti-ATXN2L and anti-ATXN2 (BD Biosciences). **B)** Cell lysates were prepared from HEK293T and HeLa cells as described in Materials and Methods. Co-immunoprecipitation experiments were carried out with an anti-ATXN2L antibody, and precipitated proteins were detected using specific antibodies against PABP, DDX6 or ATXN2 (BD Biosciences). **C)** HeLa cells were fixed and ATXN2L or ATXN2 were stained with an anti-ATXN2L antibody or an anti-ATXN2 antibody (Sigma, red). SR-splicing proteins were stained using an anti-SR antibody (green). Nuclei were visualized by Hoechst staining (blue). Scale bars represent 20 µm.

Since ATXN2L and ATXN2 share most functional motifs and domains as outlined in Fig. 1A [10], both proteins likely act in related pathways. Our earlier work demonstrated that ATXN2 interacts with PABP and with the RNA helicase DDX6/Rck [14], [15]. In this regard, the PAM2 motif, a binding motif found in proteins interacting with the PABC domain of PABP [12], is also present in ATXN2L. Moreover, the acidic domain, which comprises the LSm domain and the LSm-associated domain (LSmAD), represents the interaction surface between ATXN2 and DDX6, and is 69% identical (80% sequence similarity) in both proteins [10], [15], [25]. Accordingly, we set out to analyze whether ATXN2L is also found in association with PABP and DDX6. For this, we performed co-immunoprecipitation experiments using cell lysates prepared from HEK293T and HeLa cells. After incubation of cell lysates with an antibody directed against ATXN2L, precipitated proteins were analyzed by immunoblotting. As shown in Fig. 1B, we were able to precipitate endogenous PABP (left panel) and DDX6 (middle panel) with an ATXN2L-specific antibody from both cell lysates. Due to this finding, we additionally investigated whether ATXN2L and ATXN2 are found in association as well. Again, cell lysates were prepared from these two cell lines and processed as described. We observed that endogenous ATXN2 was precipitated with the ATXN2L-specific antibody as well (Fig. 1B, right panel). In addition, we included the neuroblastoma cell line SH-SY5Y used previously [15] and observed same results (Fig. S1A). Thus, ATXN2L is found in a complex comprising PABP, DDX6, and ATXN2 in mammalian cells indicating that ATXN2L is involved in cellular RNA processing pathways as well.

Next we investigated the intracellular localization of endogenous ATXN2L by confocal microscopy as described in Materials and Methods. This analysis revealed that ATXN2L is primarily cytoplasmic but also present in nuclear structures that co-localize with SR proteins (Fig. 1C, upper panel). These represent markers of nuclear splicing speckles [26], which belong to a family of splicing regulators with a characteristic domain rich in arginine and serine residues [27]. Thus, ATXN2L is a component of splicing speckles. Since a nuclear localization was also reported for ATXN2 [28], we further analyzed whether endogenous ATXN2 is part of these structures as well. As shown in Fig. 1C (lower panel), nuclear ATXN2 did not co-localize with SR-positive structures in HeLa cells under the chosen conditions. Thus, our findings indicate that ATXN2L is associated with the nuclear splicing machinery.

### ATXN2L is a Component of SGs

**Figure 2:**
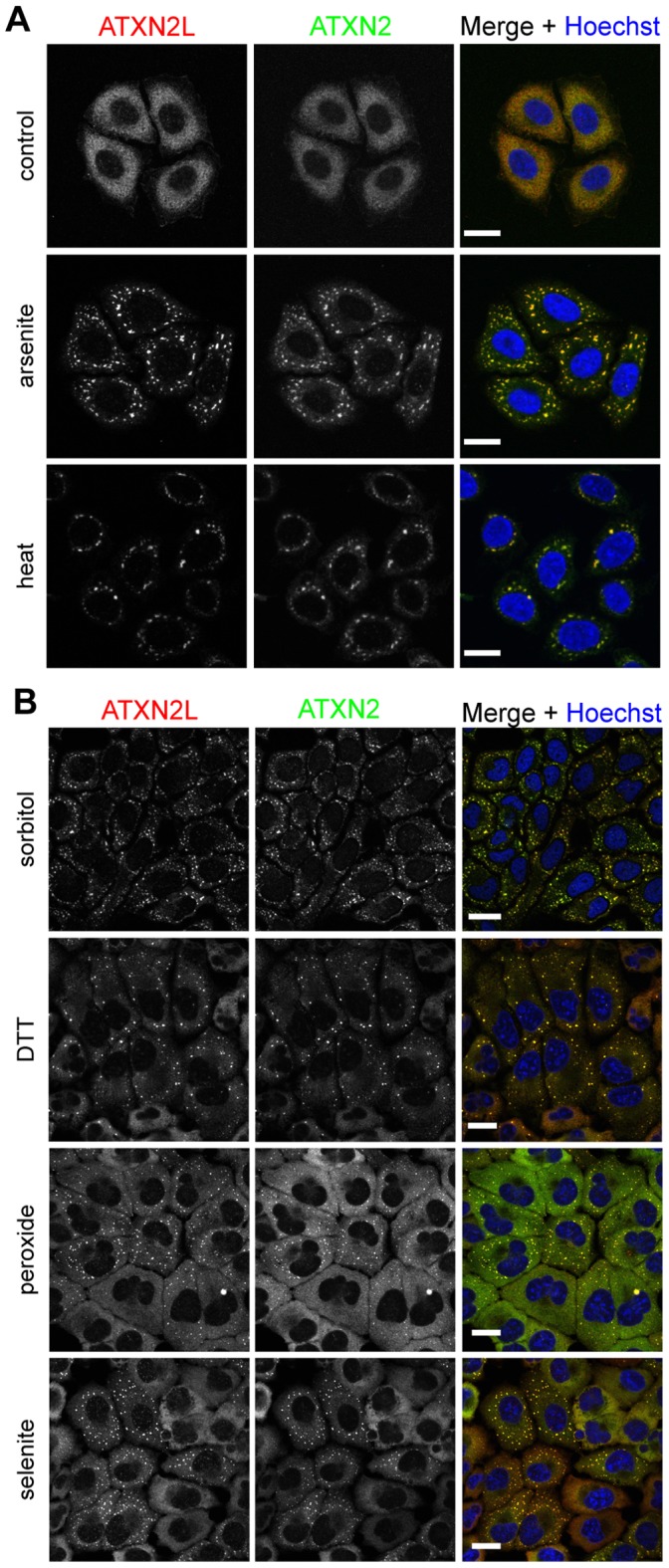
ATXN2L is a component of SGs under different stress conditions. **A)** HeLa cells were subjected to treatment with 0.5 mM sodium arsenite or heat-shock at 44°C, or **B)** 0.5 M sorbitol, 2 mM dithiothreitol (DTT), 2 mM hydrogen peroxide, or 1 mM sodium selenite for 1 hour, respectively, or left untreated as control. Cells were fixed and proteins were stained with antibodies directed against ATXN2L (red) and ATXN2 (BD Biosciences, green). Nuclei were stained with Hoechst (blue) and scale bars represent 20 µm.

**Figure 3:**
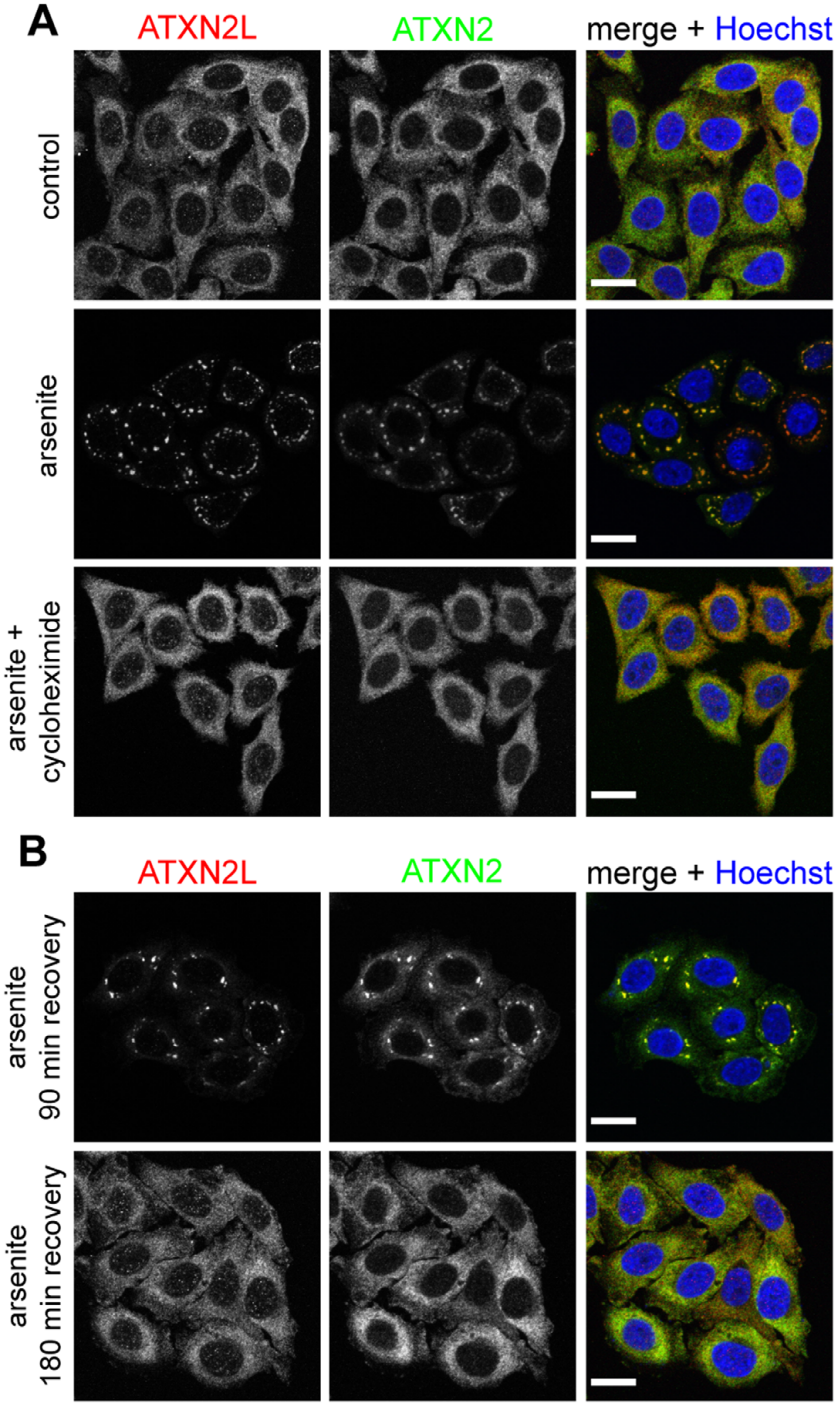
ATXN2L behaves as a dynamic SG component like ATXN2. A) HeLa cells were concurrently treated with cycloheximide and 0.5 mM sodium arsenite for 1 hour. As controls, cells were only treated with sodium arsenite, or cells were left untreated at 37°C. **B)** Cells were treated with 0.5 mM sodium arsenite for 1 hour and incubated at normal growth conditions for 90 or 180 min to allow recovery. Subsequently, cells were fixed and stained with antibodies directed against ATXN2L (red) and ATXN2 (BD Biosciences, green). Nuclei were stained with Hoechst (blue). Scale bars represent 20 µm.

Since ATXN2, DDX6, and PABP are known components of SGs [14], [15], [29], [30], we investigated in the next step whether endogenous ATXN2L is part of these cellular structures as well. To induce the formation of SGs, HeLa cells were first treated with sodium arsenite or heat-shocked. Then, cells were fixed and stained with ATXN2- and ATXN2L-specific antibodies, and the localization of both proteins was analyzed by confocal microscopy. As shown in Fig. 2A, ATXN2L localizes in distinct cytoplasmic foci in arsenite- and heat-treated cells, which were also positive for ATXN2. We also included the core SG marker proteins T-cell-restricted intracellular antigen-1-related protein (TIAR) and PABP in this analysis, and detected a co-localization of ATXN2L-positive foci with TIAR- and PABP-positive foci in arsenite- and heat-treated cells (Fig. S1B, S1C). Since the composition of SGs can vary under different stress conditions [22], [23], [31], we investigated the localization of ATXN2L under additional stresses, such as osmotic stress induced by sorbitol [32], ER stress induced by dithiothreitol (DTT) [33], and oxidative stress induced by sodium selenite or hydrogen peroxide (H_2_O_2_) [34], [35]. Also under these conditions ATXN2L formed cytoplasmic foci that co-localize with ATXN2-positive foci (Fig. 2B). Moreover, we performed stress experiments in the presence of cycloheximide, a setting preventing SG assembly [21]. For this, HeLa cells were treated with arsenite in the presence of cycloheximide, fixed, and analyzed by confocal microscopy. We observed that ATXN2L and ATXN2 retained their cytoplasmic distribution in HeLa cells with concurrent cycloheximide treatment, whereas in arsenite-treated control cells both proteins are localized to SGs (Fig. 3A). We also performed this experiment with heat-treated cells and made the same observations (Fig. S2A). Finally, we analyzed the localization of ATXN2L under conditions in which SGs disassemble [21]. Subsequent to arsenite treatment, HeLa cells were incubated for additional 90–180 min under normal growth conditions, fixed, and analyzed by confocal microscopy. As shown in Fig. 3B, ATXN2L-positive as well as ATXN2-positive foci dispersed in arsenite-treated cells after 180 min recovery and both proteins were diffusely distributed throughout the cytoplasm. Again, similar findings were obtained under heat stress (Fig. S2B). In conclusion, our results demonstrate that ATXN2L is a *bona fide* SG component.

### Altered Intracellular ATXN2L Concentration Influences SG Formation

**Figure 4:**
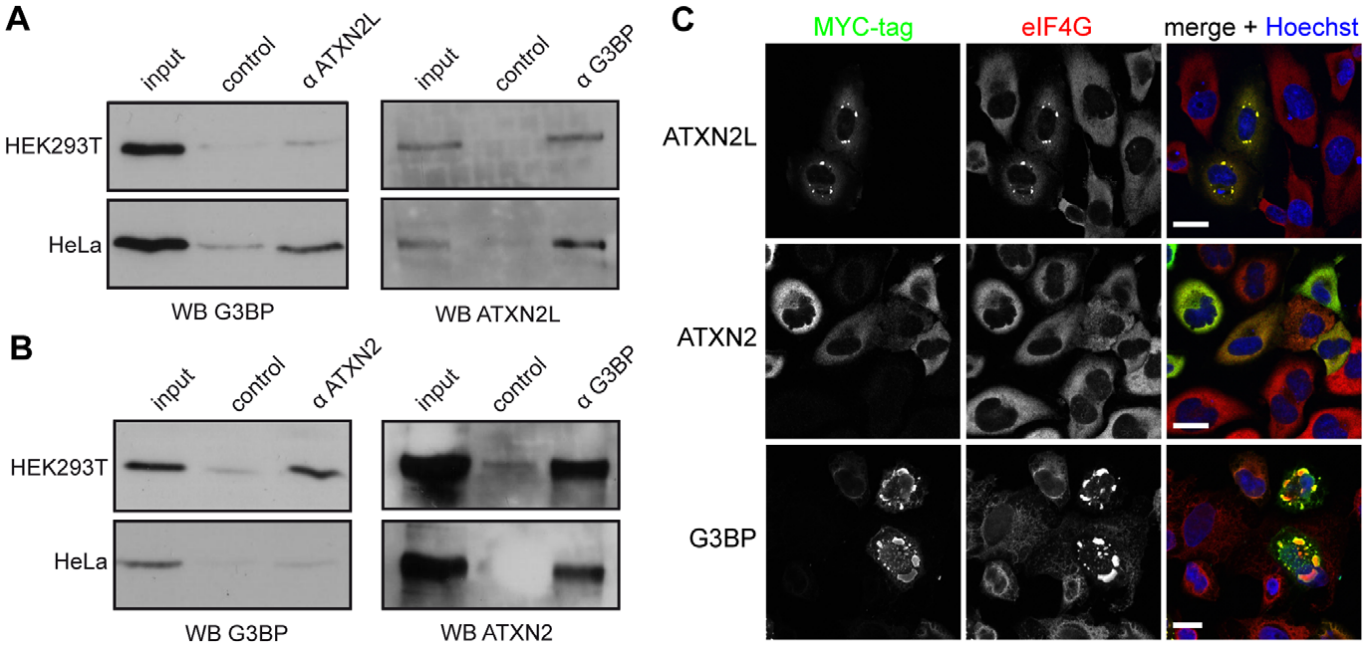
ATXN2L overexpression induces the formation of SGs. A) For co-immunoprecipitation experiments cell lysates were prepared from HEK293T and HeLa cells as described in Materials and Methods, and experiments were carried out with either anti-ATXN2L or anti-G3BP antibody. Precipitated proteins were detected with anti-G3BP or anti-ATXN2L. **B)** Co-immunoprecipitation experiments were carried out with either anti-ATXN2 (Bethyl) or anti-G3BP antibody. Precipitated proteins were detected with anti-G3BP or anti-ATXN2 (Bethyl). **C)** HeLa cells were transfected with the expression plasmids RSV-ATXN2L-MYC, pCMV-MYC-ATXN2-Q22 or pCMV-MYC-G3BP1 and incubated for 48 hours to allow expression of the respective fusion proteins. Afterward, cells were fixed and stained for the MYC-tag (Millipore, green) and eIF4G (red) to monitor induction of SGs. Nuclei were stained using Hoechst (blue). Scale bars represent 20 µm.

A high-throughput approach revealed an association between ATXN2L and the RasGAP-associated endoribonuclease G3BP [36], which if overexpressed induces the formation of SGs *per se* [37]. Accordingly, we first confirmed the described association between ATXN2L and G3BP. For this, co-immunoprecipitation experiments were carried out with HEK293T and HeLa cell lysates. As shown in Fig. 4A, we were able to precipitate endogenous G3BP with an ATXN2L-specific antibody (left panel), and endogenous ATXN2L with a G3BP-specific antibody (right panel). Moreover, we investigated whether ATXN2 is also associated with G3BP and carried out further co-immunoprecipitation experiments. We observed that endogenous G3BP was precipitated with an antibody directed against ATXN2 and *vice versa* (Fig. 4B), demonstrating complex formation between both ataxin proteins and G3BP.

Next we addressed the question whether ATXN2L overexpression may possibly induce SGs as the SG marker protein G3BP does [37]. We transfected HeLa cells with the respective plasmids for overexpressing ATXN2L, ATXN2, or G3BP, and analyzed their impact on SG induction using the SG marker protein eukaryotic translation initiation factor 4 gamma (eIF4G) in our confocal microscopy analysis. Of note, we observed that cells with ATXN2L overexpression exhibited eIF4G-positive foci, while eIF4G remained evenly distributed in non-transfected cells (Fig. 4C, upper panel). SG formation was not detected in ATXN2 overexpressing cells using eIF4G staining (Fig. 4C, middle panel). As expected, we observed that overexpressed G3BP co-localized with eIF4G-positive foci (Fig. 4C, lower panel). Thus, overexpression of ATXN2L induces SG formation.

**Figure 5:**
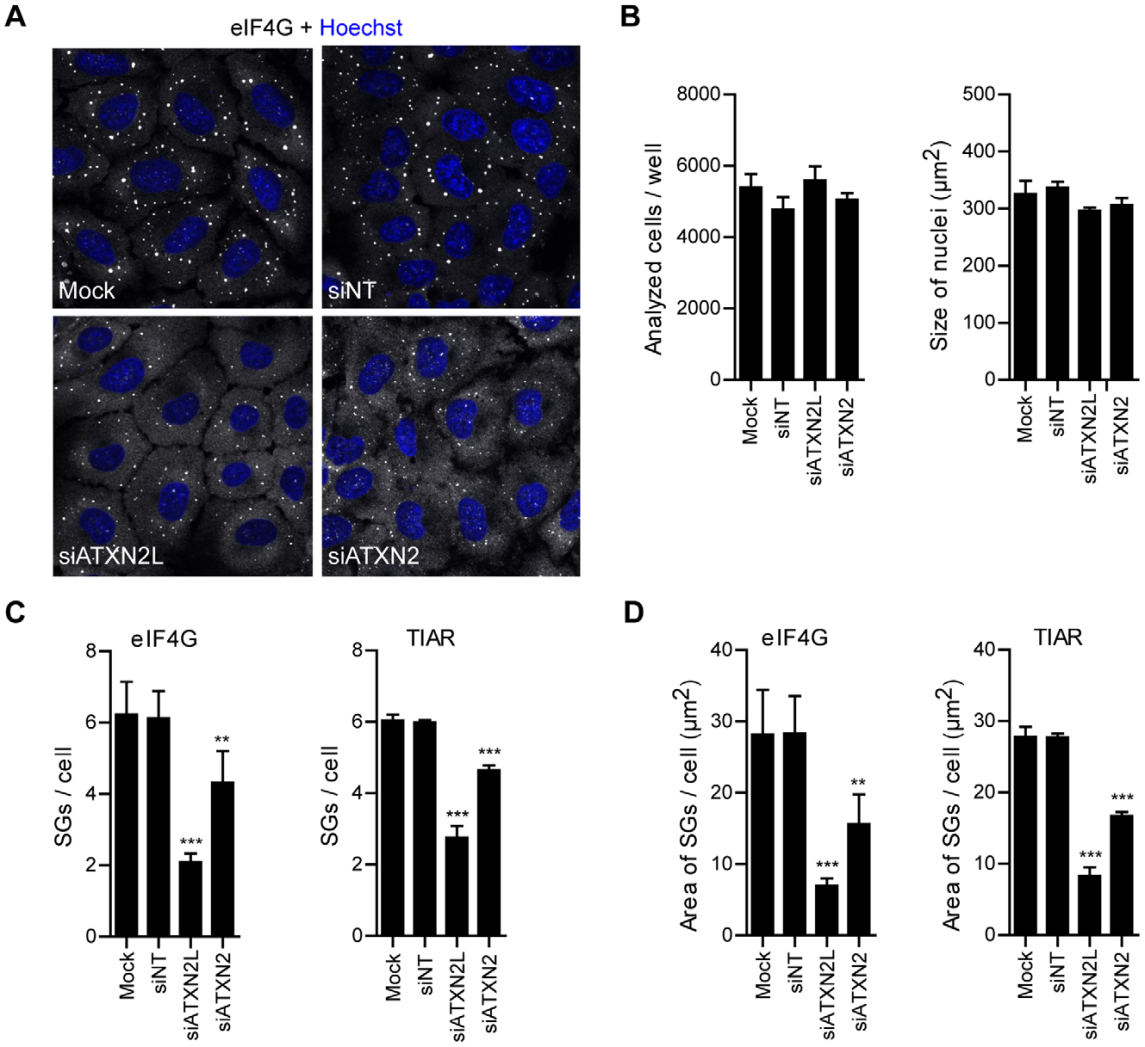
A reduced ATXN2L level affects the size and number of SGs. HeLa cells were left untreated (mock) or transfected with siRNAs against ATXN2L or ATXN2 transcripts, or non-targeting control siRNA (siNT). 72 hours post transfection cells were treated with 0.5 mM sodium arsenite for 1 hour and fixed. **A)** For the confocal microscopy proteins were stained with an antibody detecting eIF4G. (**B-D**) For the quantitative high-content screening microscopy staining occurred with antibodies detecting eIF4G and TIAR. **B)** Cell number and size of nuclei was analyzed by quantitative high-content screening microscopy. **C, D)** Quantification of TIAR- and eIF4G-positive SGs regarding number and size. Results are expressed as mean ± SD from one representative experiment, n = 5 replicate wells, *p<0.05; **p<0.01; ***p<0.001, One-way ANOVA with Tukey’s Multiple Comparison post test.

In this regard, we showed earlier that reduction of the intracellular ATXN2 level has an impact on SG formation [15]. Consequently, we analyzed the influence of a reduced ATXN2L level on SG formation as well. First we verified the ATXN2L or ATXN2 knock down by immunoblotting and microscopy (Fig. S3). Then, we transfected HeLa cells with specific siRNA molecules targeting ATXN2L or ATXN2 transcripts, with unspecific non-targeting (NT) siRNA molecules, or left cells untreated (mock), and exposed cells to arsenite stress 72 h after transfection. After fixation the SG marker proteins eIF4G and TIAR were stained and analyzed by confocal microscopy. As shown in Fig. 5A, cells with reduced ATXN2L or ATXN2 level exhibited fewer and smaller eIF4G-positive foci compared to control cells (mock and siNT). To further corroborate and quantify these findings, we additionally performed an automated microscopy approach based on a Cellomics ArrayScan VTI high-content screening platform. This system automatically acquires images of stained cells in multi-well plates. Cells are identified by nuclear staining and fixed object selection parameters, and SGs are quantified within a circular area extending the nuclear region (Fig. S4; for details please see Materials and Methods). First, we excluded that transfection of siRNA molecules has an impact on cell survival or the nuclear size representing a basic morphological parameter (Fig. 5B). Of note, we observed that in cells with a lowered ATXN2L level the number of eIF4G- and TIAR-positive SGs was significantly reduced to 34±4% or 46±5% compared to the non-targeting control (p<0.001, n = 5 replicate wells/condition, ± SD) (Fig. 5C and S4). As expected, cells with a reduced ATXN2 level also exhibited fewer eIF4G- and TIAR-positive SGs (Fig. 5C and S4). In addition, the size of SGs was considerably smaller in cells with reduced ATXN2L or ATXN2 level compared to controls (Fig. 5D). Similar results were obtained if G3BP was used as SG marker protein (data not shown). Thus, our findings demonstrate that ATXN2L is important for SG formation.

### ATXN2L Level Influences P-body Formation

**Figure 6:**
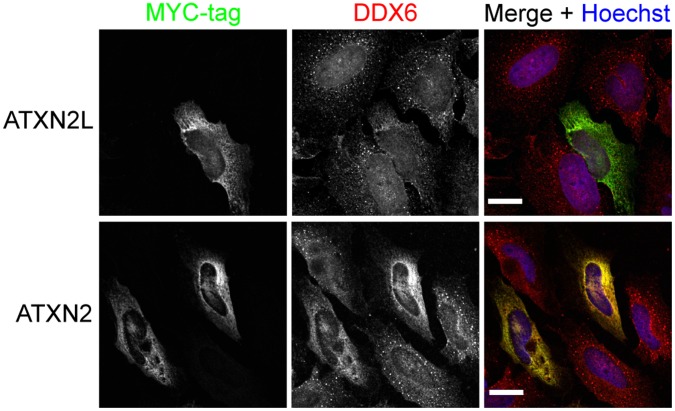
P-body formation is influenced by ATXN2L overexpression. HeLa cells were transfected with expression constructs RSV-ATXN2L-MYC or pCMV-MYC-ATXN2-Q22 to overexpress ATXN2L or ATXN2, respectively. 48 h post transfection, cells were fixed and stained with antibodies directed against the MYC-tag to visualize ATXN2L or ATXN2 overexpressing cells and an antibody against DDX6. Nuclei were stained with Hoechst. Images shown are taken from one representative experiment. Scale bars represent 20 µm.

We reported earlier that overexpression of ATXN2 influences the presence of microscopically visible P-bodies in HEK293T cells, whereas no obvious effect was observed in case ATXN2 levels were reduced [15]. Consequently, we wanted to analyze the effect of altered ATXN2L levels on P-body formation as well. First, HeLa cells were transfected with the expression constructs RSV-ATXN2L-MYC or pCMV-MYC-ATXN2-Q22 to overexpress ATXN2L or ATXN2, respectively. Cells were then incubated for 48 h to allow expression of proteins, fixed and treated with an antibody directed against the MYC-tag to visualize cells overexpressing ATXN2L or ATXN2. For visualization of P-bodies an antibody directed against the component DDX6 was used. As shown in Fig. 6, cells overexpressing ATXN2L exhibited a diffuse cytoplasmic localization of DDX6, whereas in non-transfected cells DDX6 localized to P-bodies. As reported earlier [15], a comparable reduction in P-body number was observed in cells overexpressing ATXN2 (Fig. 6). Thus, overexpression of ATXN2L affects the presence of microscopically visible P-bodies.

**Figure 7:**
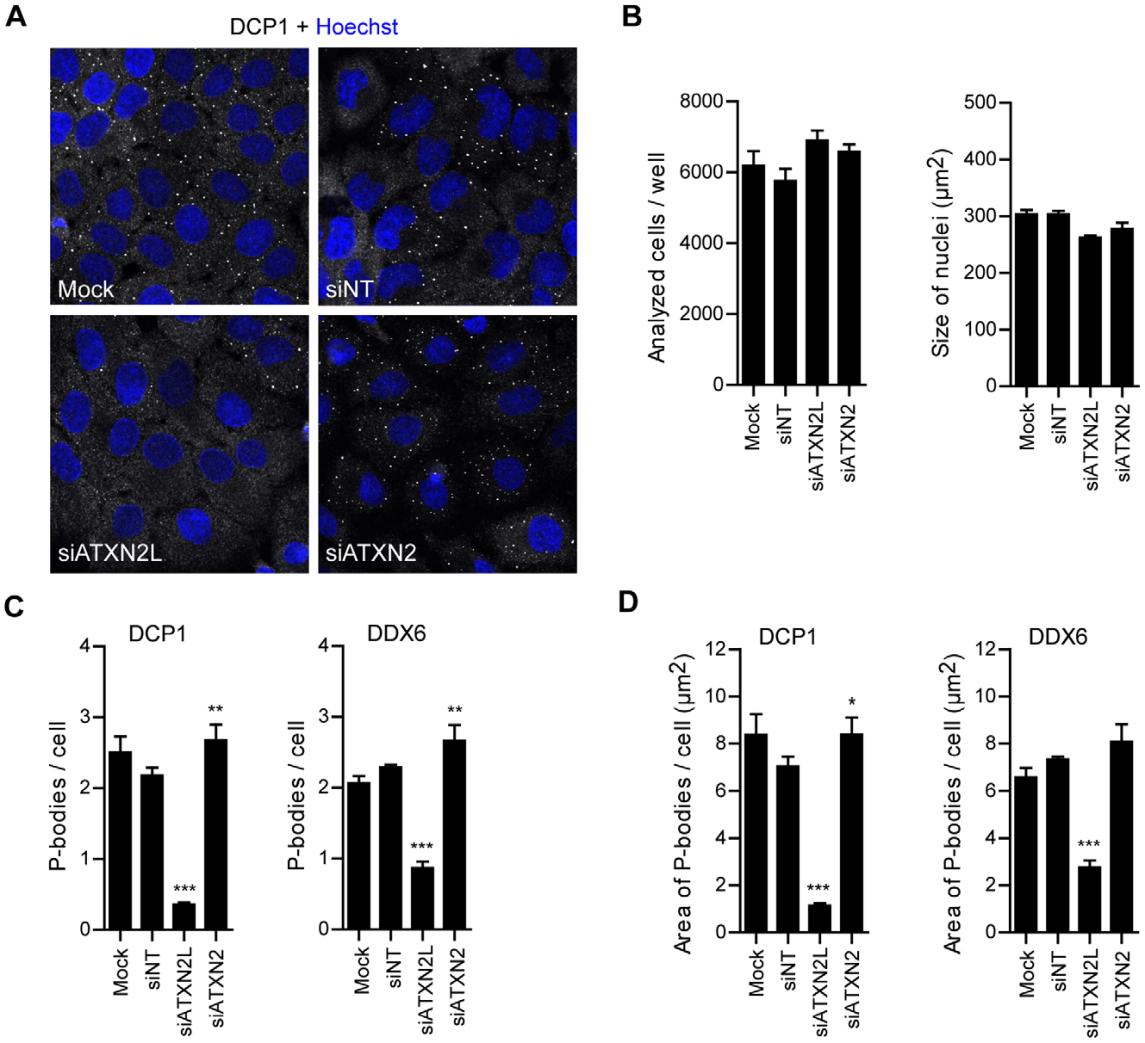
ATXN2L reduction affects P-body formation. HeLa cells were left untreated (mock) or transfected with siRNAs against ATXN2L or ATXN2 transcripts, or non-targeting control siRNAs. The cells were fixed 72 hours post transfection, and analyzed **A)** by confocal microscopy or **B–D)** by quantitative high-content screening microscopy. In **A)** cells were stained with an antibody detecting DCP1. In **B–D)** cells were stained with antibodies detecting DCP1 and DDX6. **B)** Cell numbers and size of nuclei of analyzed cells. **C, D)** Quantification of DCP1- and DDX6-positive P-bodies regarding number and size. Results are expressed as mean ± SD from one representative experiment, n = 5 replicate wells, *p<0.05; **p<0.01; ***p<0.001, One-way ANOVA with Tukey’s Multiple Comparison post test.

We then set out to investigate the effect of a lowered intracellular ATXN2L level on P-body formation. For this, HeLa cells were transfected with specific siRNA molecules for ATXN2L or ATXN2 or with unspecific non-targeting siRNA molecules, and P-body formation was analyzed by confocal and automated high-content screening microscopy. Confocal microscopy revealed that the number of DCP1-positive P-bodies was strongly reduced in cells with low ATXN2L level compared to control cells treated with non-targeting molecules (Fig. 7A). Moreover, no obvious effect on P-body number was detected in cells with reduced ATXN2 level, which is in consistency with our earlier study using HEK293T cells [15]. For the quantification of P-bodies using our automated microscopy approach we again first determined that a similar number of cells was analyzed, and that no effect occurred on the nuclear size (Fig. 7B). Interestingly, this approach revealed that in cells with reduced ATXN2L level the number of DCP1- and DDX6-positive P-bodies was decreased to 16±1% and 38±4% compared to the non-targeting control, and the size of P-bodies was reduced as well (Fig. 7C, D and S5). We also detected a minor increase in the number and size of microscopically visible P-bodies in cells with low ATXN2 level. In sum, ATXN2L is a regulator of P-body formation.

## Discussion

In this study, we revealed a functional overlap between ATXN2L and ATXN2 with regard to RNA metabolism, since ATXN2L associates with known interaction partners of ATXN2, the RNA helicase DDX6 and PABP [14], [15], and with ATXN2 itself. Moreover, we discovered that ATXN2L is a *bona fide* component of SGs in mammalian cells under different stress conditions. Most importantly, we identified ATXN2L as a regulator of SGs, since ATXN2L overexpression caused induction of SGs, whereas a low ATXN2L level reduced the number and size of SGs. The overexpression or reduction of several proteins have been reported to affect SG formation or SG composition [38], possibly at different steps, since the process of SG formation has been categorized in different stages [22]. The first stage begins with stalled initiation and ribosome runoff marking ribonucleoprotein complexes as sites where SGs assemble. This stage is followed by the primary aggregation or SG nucleation step initiated by RNA-binding proteins with aggregation-prone properties, such as T-cell-restricted intracellular antigen-1 (TIA-1) and G3BP [22], [37], [39], followed by the secondary aggregation step. Finally, proteins are recruited through protein-protein interactions [22]. Of note, we observed an association between G3BP and ATXN2L in HeLa cells that has been originally described in a high-throughput analysis [36], suggesting that ATXN2L could be important for the nucleation step as G3BP is. Interestingly, we also detected an association between ATXN2 and G3BP in this study. Even so, we observed that ATNX2 overexpression has no effect on SG induction in HeLa cells under the chosen experimental settings, whereas a reduced ATXN2 level also affects number and size of SGs, consistent with our earlier study using HEK293T cells [15].

Evidence has been provided that posttranslational modifications of proteins are important in the complex dynamic process of SG assembly. Many RNA-binding proteins implicated in the primary nucleation step contain methylatable domains [40], [41]. For the fragile × mental retardation protein (FMRP), the methylation of its RRG rich domain is important for its function as inducer of SGs [42]. Remarkably, an arginine methylation site at position 361 in the ATXN2L protein has been identified to be methylated, whereas this amino acid position is not conserved in the ATXN2 protein [43]. Interestingly, the overexpression of a phosphomimetic G3BP mutant failed to assemble SGs, while overexpression of a nonphosphorylatable G3BP mutant caused SG formation [37]. Therefore, it can be speculated that differences in posttranslational modifications between ATXN2L and ATXN2 are accountable for the observed results regarding SG formation, a task that will be addressed in the future.

On the other hand, we discovered that ATXN2L is a regulator of P-bodies as well, since ATXN2L overexpression and reduction decreases the number and size of P-bodies in HeLa cells, whereas only ATXN2 overexpression had a noticeable effect on P-bodies as reported earlier [15]. Analogous to the assembly of SGs, P-body formation is also affected by the overexpression and reduction of various P-body components [24]. Interestingly, depletion of DDX6 results in the loss of microscopically visible P-bodies [44]. We speculated in our earlier study that the observed mis-localization of DDX6 and loss of microscopically visible P-bodies in cells overexpressing ATXN2 might be based on abnormal protein interactions attributable to the increase in the interaction surface, the LSm/LSmAD domain of ATXN2 [15]. Since this domain is conserved in the ATXN2L protein [10], such a recruitment mechanism is likely to explain the observed effect as well. Regarding the effect on P-body number and size observed in cells with a reduced ATXN2L concentration, it might be of interest that ATXN2L comprises a sequence in the C-terminal region that shows homology to the Pat1 protein family, which is absent in the ATXN2 protein. The Pat1 protein family is conserved in eukaryotes and two Pat1 proteins, PatL2/Pat1a and PatL1/Pat1b, exist in humans [45], [46]. PatL1/Pat1b is a P-body component and the expression of PatL1/Pat1b protein lacking certain domains or its depletion results in loss of microscopically visible P-bodies, whereas PatL1/Pat1b overexpression induces P-body formation [46], [47]. Moreover, PatL1/Pat1b shuttles between the cytoplasm and nucleus, and nuclear PatL1/Pat1b localizes to splicing speckles, indicating that it is implicated in RNA-related processes in both cellular compartments [48]. In this light, it is interesting that we observed a co-localization of ATXN2L and nuclear splicing speckles, suggesting that ATXN2L may function in splicing processes as well. On the other hand, localization of splicing factors is regulated by protein arginine methylation, which also modulates associations between SR-like proteins and splicing factors [49], [50]. Nonetheless, lysine acetylation is another posttranslational modification that controls the localization and association of proteins involved in various cellular processes including amongst others gene transcription, translation or splicing [51]. A high-resolution mass spectrometry approach identified ATXN2L as target for lysine acetylation, while ATXN2 was not detected [52]. Again, such a difference could be responsible for the observed effects on P-body formation as well.

Further studies are necessary to dissect and define the function of ATXN2L in the cellular mRNA metabolism. Moreover, it will be interesting to further explore the functional interplay between ATXN2L and ATNX2. As mentioned before, functional studies of ATXN1L/Boat, the paralog of the disease-causing protein ATXN1, revealed a suppressive activity in SCA1 disease pathogenesis [5]. Utilizing a SCA1 *Drosophila* model, Mizutani and colleagues showed that an eye defect, caused by mutant ATXN1, was suppressed by ATXN1L overexpression due to the association of both proteins [53]. Moreover, evidence was provided that the activity of mutant ATXN1 causative for the observed neurotoxic events in a SCA1 knock-in mouse model can be ameliorated by duplication of *ATXN1L* modifying incorporation of mutant ATXN1 into native protein complexes [5]. Consequently, it will be interesting to explore whether gene dosage compensation or antagonistic behavior between ATXN2L and ATXN2 might occur in SCA2 transgenic animal models and whether and how this impacts SCA2 pathogenesis.

## Materials and Methods

### Plasmids

The plasmids RSV-ATXN2L-MYC and pCMV-MYC-ATXN2-Q22 were described earlier [11], [15]. To generate plasmid pCMV-MYC-G3BP1, a PCR was carried out using human fetal brain cDNA library (Clontech) as DNA template and primer pair G3BP1-s-SalI (5′-CGAGGTCGACGGAGAAGCCTAGTCCCCTG-3′) and G3BP1-as-NotI (5′-CTGCGGCCGCTCACTGCCGTGGCGCAAG-3′). Afterwards, the amplified DNA fragment was purified, treated with *Sal*I and *Not*I, subcloned into the *Sal*I/*Not*I sites of the expression vector pCMV-MYC (Clontech), and validated by sequencing.

### Cell Cultivation and Transfection

HEK293T, HeLa and SH-SY5Y cells were cultivated in DMEM (Dulbecco’s modified Eagle medium, Invitrogen) supplemented with 100 units/ml Penicillin/G-Streptomycin (Biochrom) and 10% fetal bovine serum (FBS, Biochrom) at 37°C and 5% CO_2_. Transfections were carried out in 24-well plates using 1–2 µg RSV-ATXN2L-MYC, pCMV-MYC-ATXN2-Q22, or pCMV-MYC-G3BP1 and 3–6 µl Polyethylenimine (PEI, linear, MW 25 kDa, 1 mg/ml, pH 7, Polysciences Inc.), respectively. Then, transfected cells were incubated for 48 hours to allow transient expression of proteins.

### Co-immunoprecipitation

Co-immunoprecipitation experiments were performed as described [15]. Briefly, HEK293T, HeLa or SH-SY5Y cells were washed in PBS, harvested and lysed in lysis buffer [20 mM Tris-HCl, pH 7.4, 150 mM NaCl, 1 mM EDTA, 1% Triton-X100, 2.5% Protease-inhibitor (“complete” tablets, Roche), and 25 units/ml benzonase (Merck)] for 30 minutes at 4°C. 300–500 µg of each cell lysate was incubated with 1.5 µl primary antibody [rabbit anti-ATXN2L (Bethyl A301-370A), rabbit anti-ATXN2 (Bethyl A301-118A) or mouse anti-G3BP (Abnova)] overnight at 4°C. Then, 15–20 µl IgG conjugated M-280 Dynabeads (Dynal) were added and samples were incubated for additional 2–3 hours. Dynabeads were pulled down magnetically and washed three times with 3% BSA/PBS and 3 times with PBS. Finally, SDS sample buffer containing 0.1 M DTT was added, samples were incubated at 95°C for 5 min, and loaded onto a 10% SDS gel. After separation, proteins were transferred to a PVDF membrane (Millipore) using a semi-dry blotting system (PeqLab). Subsequently, membranes were incubated overnight with primary antibodies as indicated [rabbit anti-ATXN2L (Bethyl, 1∶1000), mouse anti-ATXN2 (BD Biosciences, 1∶1000), rabbit anti-ATXN2 (Bethyl, 1∶1000), mouse anti-DDX6 (Abnova, 1∶2000), mouse anti-PABP (Abcam, 1∶1000) and mouse anti-G3BP (Abnova, 1∶1000)]. Then, membranes were incubated with secondary antibodies [POD-conjugated anti-mouse or anti-rabbit, 1∶10000 (Sigma)] for 1 hour and proteins were visualized using Western Lightning ECL solutions (Perkin Elmer).

### RNA Interference Experiments

HeLa cells were seeded in 24-well plates on glass slides in DMEM supplemented with 10% FBS for 24 hours. Then, 1.2 µl 20 µM siRNA molecules [ATXN2L Stealth Select RNAi Pool (Invitrogen), ATXN2 On Target Plus Smart Pool, On Target Plus Non-targeting Pool (Dharmacon)] and 3 µl Lipofectamine RNAiMAX transfection reagent (Invitrogen) were mixed, incubated for 10 min and added to the cells. As mock control, untreated cells were included. 72 hours post transfection cells were exposed to oxidative stress by treatment with 0.5 mM sodium arsenite for 1 hour or left untreated. Afterward, cells were fixed with 2% formaldehyde for 10 min and ice-cold methanol for at least 30 min and processed for microscopic analyses as described [15]. For automated microscopy, HeLa cells were seeded in 6-well plates, treated with siRNA concentrations as described above and incubated for 72 hours. Then, cells were treated with trypsin, seeded in 96-well imaging plates (Greiner µClear) for 12–24 hours, and exposed to arsenite-stress or left untreated. For validation of knock down, HeLa cells were seeded in 12-well plates and treated with siRNA concentrations as described above and incubated for 72 hours. Cells were then lysed and proteins were subjected to SDS-PAGE and Western blotting as described above and detected using rabbit anti-ATXN2L (Bethyl, 1∶1000), mouse anti-ATXN2 (BD Biosciences, 1∶1000), mouse anti-G3BP (Abnova, 1∶1000) and mouse anti-GAPDH (Ambion, 1∶5000) antibodies. Equal protein loading was confirmed by Coomassie staining.

### Confocal Microscopy

For stress experiments, cells were seeded on glass slides and either incubated with 0.5 mM sodium arsenite, 0.5 M sorbitol, 2 mM dithiothreitol (DTT), 2 mM hydrogen peroxide (H_2_O_2_), 1 mM sodium selenite (Sigma) or exposed to heat stress at 44°C for 1 hour, while control cells were left untreated. To analyze the effect of cycloheximide on ATXN2L localization, 10 µg/ml cycloheximide was added to arsenite or heat-treated cells. For recovery experiments, medium was removed after the chosen stress conditions, fresh medium was applied, and cells were incubated for additional 90–180 min. Cells were then fixed and proteins were stained with the respective primary antibodies [rabbit anti-ATXN2L (1∶300, Bethyl), mouse anti-ATXN2 (1∶200, BD Biosciences), rabbit anti-ATXN2 (1∶200, Sigma HPA020339); mouse anti-DCP1A (1∶400, Abnova), rabbit anti-DDX6 (Novus Biologicals, 200-192), rabbit anti-eIF4G1 (1∶200, Abcam), mouse anti-MYC (1∶500, Millipore), mouse anti-PABP (1∶100, Abcam), mouse anti-SR proteins (1∶200; Invitrogen) and mouse anti-TIAR (1∶200, BD Biosciences)] in 3% BSA/PBS for 1 hour at room-temperature. After addition of the respective secondary antibodies [anti-rabbit Alexa Fluor 594 (1∶500, Invitrogen), anti-mouse Alexa Fluor 488 (1∶500, Invitrogen)], nuclei were stained with bisbenzimide (Hoechst, Sigma) and samples were mounted with Fluoromount-G (Southern Biotech). Cells were analyzed using a confocal microscope (LSM 700, Zeiss) on an inverted stand (Axiovert 200 M, Zeiss) using objective Plan-NEOFLUAR 40×1.3 oil DIC. Images were prepared using Zeiss software ZEN version 5.5.

### High-content Screening Microscopy

Fixed cells in 96-well imaging plates were stained with the respective primary antibodies [mouse anti-TIAR (1∶200, BD Biosciences), rabbit anti-eIF4G1 (1∶200, Abcam), mouse anti-DCP1A (1∶400, Abnova), rabbit anti-DDX6 (Novus Biologicals, 200-192)] and secondary antibodies [anti-rabbit Alexa Fluor 594 (1∶500, Invitrogen), anti-mouse Alexa Fluor 488 (1∶500, Invitrogen)] in 3% BSA/PBS for 1 hour at room-temperature and nuclei were stained with DAPI (Sigma). Plates were scanned using a Thermo Fisher Cellomics ArrayScan VTI. Images of 512×512 pixels were acquired with a 20×objective and analyzed using the Cellomics software package (Colocalization V.4 Bioapplication). Cell nuclei were identified by DAPI staining and according to the object identification parameters size: 100–1200 µm^2^, ratio of perimeter squared to 4π area: 1–2, length-to-width ratio: 1–2, average intensity: 50–1000, total intensity: 3×10^4^−2×10^7^. SGs and P-bodies were identified within a circular region extending the nucleus by maximally 20 µm. The object identification parameters for SGs and P-bodies were: 1.5–20 µm^2^, ratio of perimeter squared to 4π area: 1–1.8, length-to-width ratio: 1–1.8, average intensity: 100–1500, total intensity: 5×10^3^–5×10^4^.

## Supporting Information

Figure S1 **ATXN2L interacts and co-localizes with different SG marker proteins. A)** Cell lysates were prepared from SH-SY5Y cells and co-immunoprecipitation experiments were carried out with an anti-ATXN2L antibody. Precipitated proteins were detected using specific antibodies against PABP, DDX6 or ATXN2 (BD Biosciences). **B, C)** HeLa cells were treated with 0.5 mM sodium arsenite or heat-shocked at 44°C for 1 hour with control cells left untreated at 37°C. Afterward, cells were fixed and stained with the corresponding antibodies to visualize ATXN2L (red) and **B)** TIAR (green) or **C)** PABP (green), respectively. Hoechst staining (blue) was used for the detection of nuclei. Scale bars correspond to 20 µm.
(TIF)

Figure S2 **ATXN2L behaves as a dynamic SG component under heat stress. A)** HeLa cells were heat-shocked in the presence of cycloheximide at 44°C for 1 hour. Heat-shocked cells or cells left untreated at 37°C served as controls. **B)** HeLa cells were heat-shocked at 44°C for 1 hour and incubated at normal growth conditions for 90 or 180 min to allow recovery. Subsequently, cells were fixed and stained with antibodies directed against ATXN2L (red) and ATXN2 (BD Biosciences, green). Nuclei were stained with Hoechst (blue). Scale bars correspond to 20 µm.
(TIF)

Figure S3 **Efficiency and specificity of ATXN2L and ATXN2 knock-down. A)** HeLa cells were left untreated (mock) or transfected with non-targeting (siNT), or ATXN2L- or ATXN2-specific siRNAs, lysed 72 hours post transfection and subjected to SDS-PAGE. Protein level of ATXN2L, ATXN2 (BD Biosciences), G3BP and GAPDH was analyzed using the corresponding antibodies. To show loading of equal amounts of protein, gel was stained using Coomassie blue. **B)** HeLa cells were transfected with non-targeting (siNT), or ATXN2L- or ATXN2- specific siRNA molecules, fixed 72 hours post transfection and stained with antibodies directed against ATXN2L (red) and ATXN2 (BD Biosciences, green).
(TIF)

Figure S4 **Quantification of TIAR- and eIF4G-positive SGs.** HeLa cells were left untreated (mock) or transfected with non-targeting (siNT), or ATXN2L- or ATXN2-specific siRNA molecules. 72 h post transfection cells were treated with 0.5 mM sodium arsenite for 1 hour to induce SG formation, fixed, and stained with TIAR- and eIF4G-specific antibodies. Cell nuclei were stained with DAPI. Representative view fields of the automated image analysis are shown. Green encircled nuclei were selected; red encircled nuclei were rejected by the object identification algorithm. Outer cell borders (blue lines) were calculated by extending the nuclear region. TIAR-positive (yellow) and eIF4G-positive (magenta) SGs were quantified within whole cells.
(TIF)

Figure S5 **Quantification of DCP1- and DDX6-positive P-bodies.** HeLa cells were left untreated (mock) or transfected with non-targeting (siNT), ATXN2L- or ATXN2-specific siRNA molecules. 72 h post transfection cells were fixed and stained with DCP1- and DDX6-specific antibodies. Cell nuclei were stained with DAPI. Representative view fields of the automated image analysis are shown. Green encircled nuclei were selected; red encircled nuclei were rejected by the object identification algorithm. Outer cell borders (blue lines) were calculated by extending the nuclear region. DCP1-positive (yellow) and DDX6-positive (magenta) P-bodies were quantified within whole cells.
(TIF)
