# Supplementary figures and images for: Ataxin-2-Like Is a Regulator of Stress Granules and Processing Bodies
Source: PLoS One. 2012 Nov 27;7(11):e50134. doi: 10.1371/journal.pone.0050134 (PMC3507954; doi:10.1371/journal.pone.0050134)

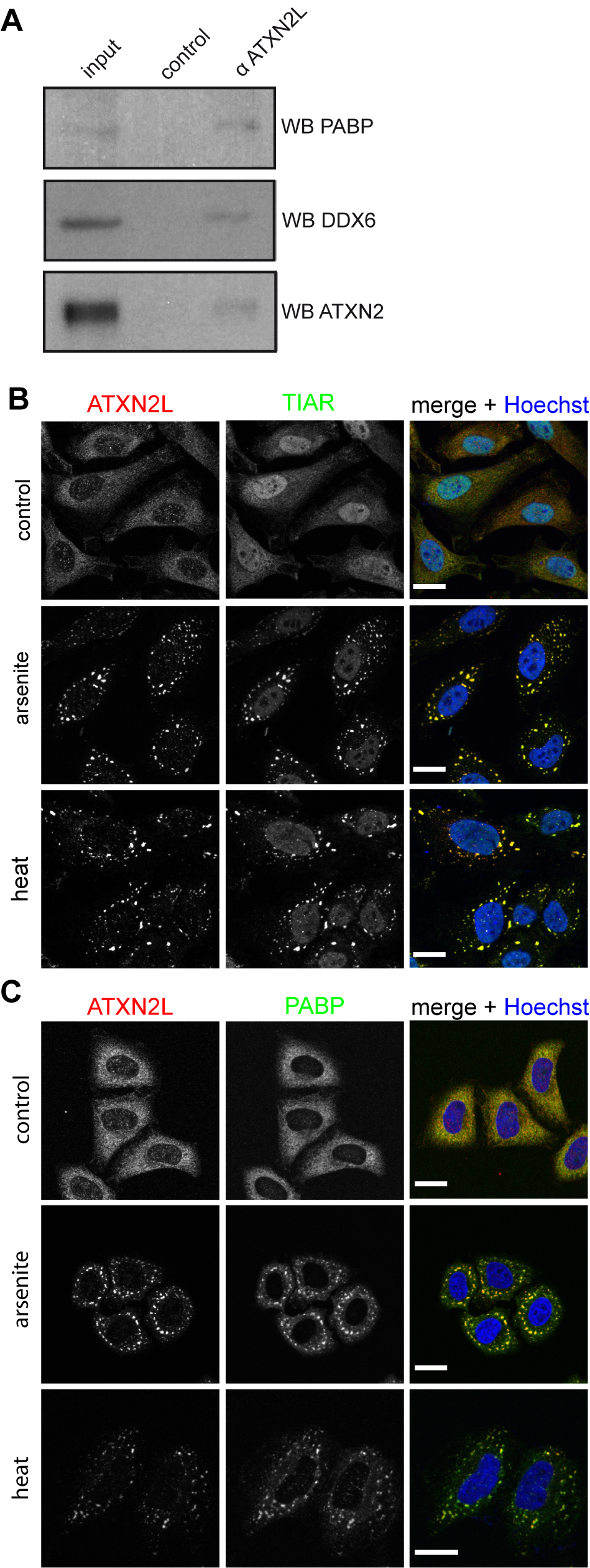

Supplement: Figure S1 — ATXN2L interacts and co-localizes with different SG marker proteins. A) Cell lysates were prepared from SH-SY5Y cells and co-immunoprecipitation experiments were carried out with an anti-ATXN2L antibody. Precipitated proteins were detected using specific antibodies against PABP, DDX6 or ATXN2 (BD Biosciences). B, C) HeLa cells were treated with 0.5 mM sodium arsenite or heat-shocked at 44°C for 1 hour with control cells left untreated at 37°C. Afterward, cells were fixed and stained with the corresponding antibodies to visualize ATXN2L (red) and B) TIAR (green) or C) PABP (green), respectively. Hoechst staining (blue) was used for the detection of nuclei. Scale bars correspond to 20 µm. (TIF) [file pone.0050134.s001.tif]

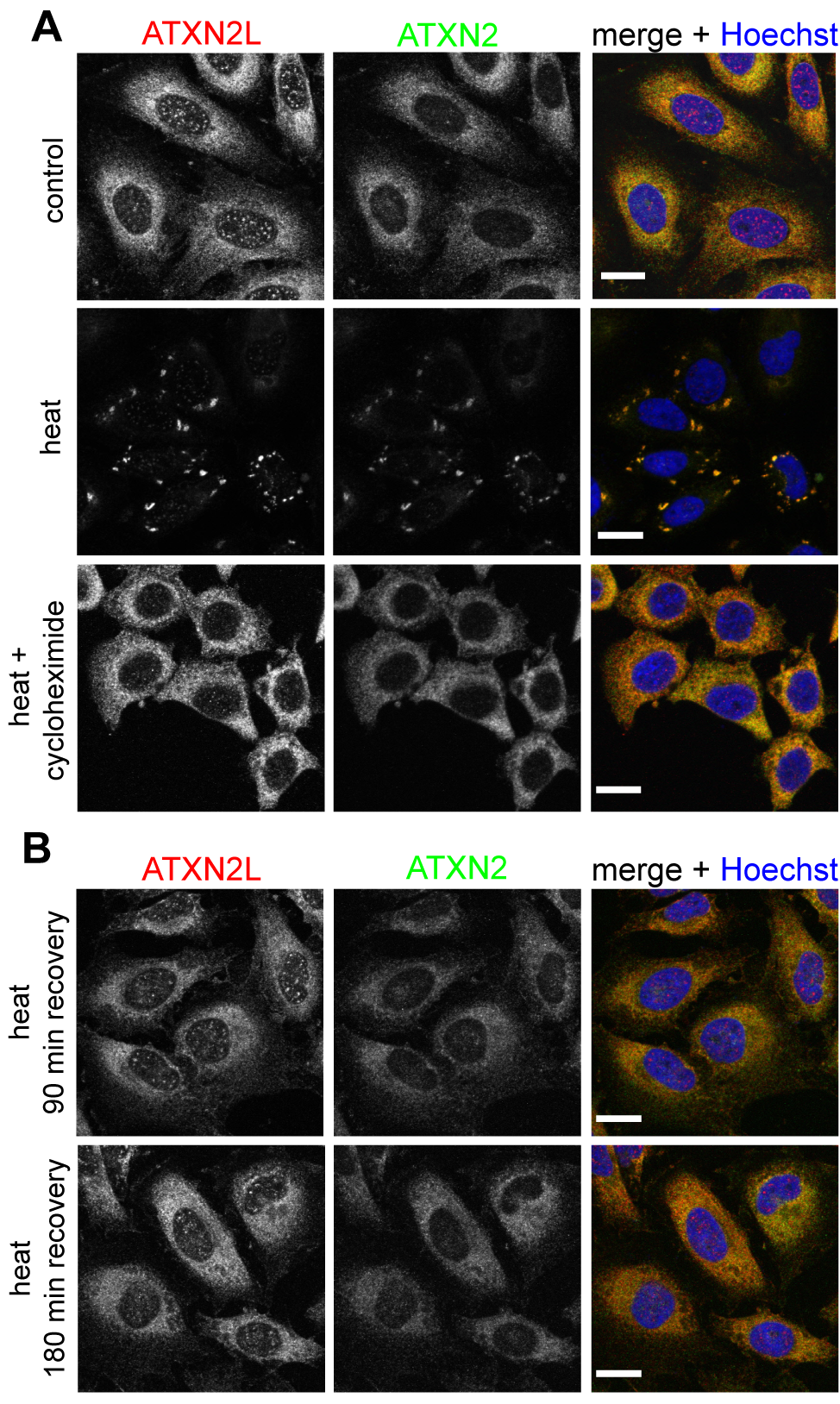

Supplement: Figure S2 — ATXN2L behaves as a dynamic SG component under heat stress. A) HeLa cells were heat-shocked in the presence of cycloheximide at 44°C for 1 hour. Heat-shocked cells or cells left untreated at 37°C served as controls. B) HeLa cells were heat-shocked at 44°C for 1 hour and incubated at normal growth conditions for 90 or 180 min to allow recovery. Subsequently, cells were fixed and stained with antibodies directed against ATXN2L (red) and ATXN2 (BD Biosciences, green). Nuclei were stained with Hoechst (blue). Scale bars correspond to 20 µm. (TIF) [file pone.0050134.s002.tif]

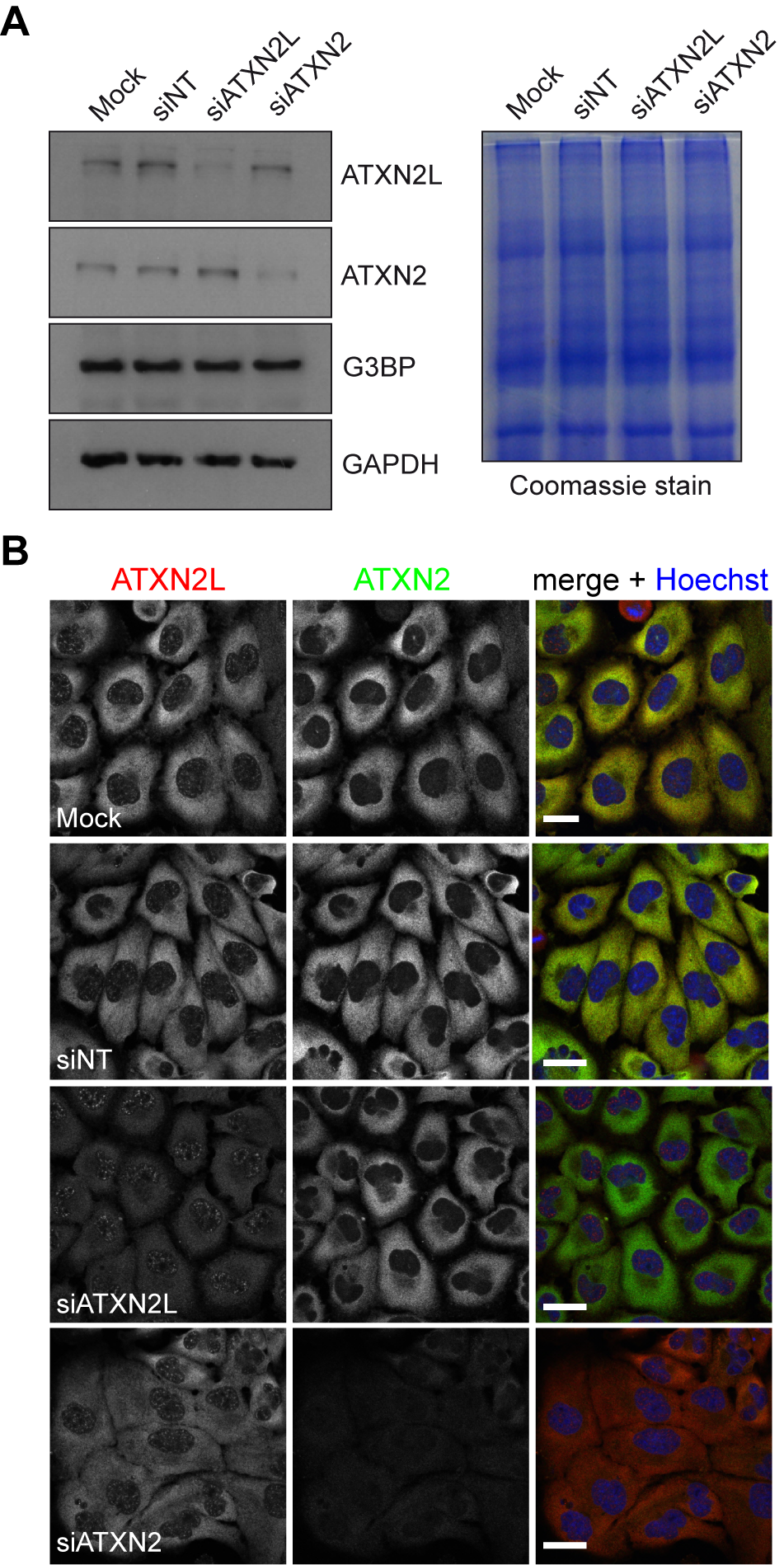

Supplement: Figure S3 — Efficiency and specificity of ATXN2L and ATXN2 knock-down. A) HeLa cells were left untreated (mock) or transfected with non-targeting (siNT), or ATXN2L- or ATXN2-specific siRNAs, lysed 72 hours post transfection and subjected to SDS-PAGE. Protein level of ATXN2L, ATXN2 (BD Biosciences), G3BP and GAPDH was analyzed using the corresponding antibodies. To show loading of equal amounts of protein, gel was stained using Coomassie blue. B) HeLa cells were transfected with non-targeting (siNT), or ATXN2L- or ATXN2- specific siRNA molecules, fixed 72 hours post transfection and stained with antibodies directed against ATXN2L (red) and ATXN2 (BD Biosciences, green). (TIF) [file pone.0050134.s003.tif]

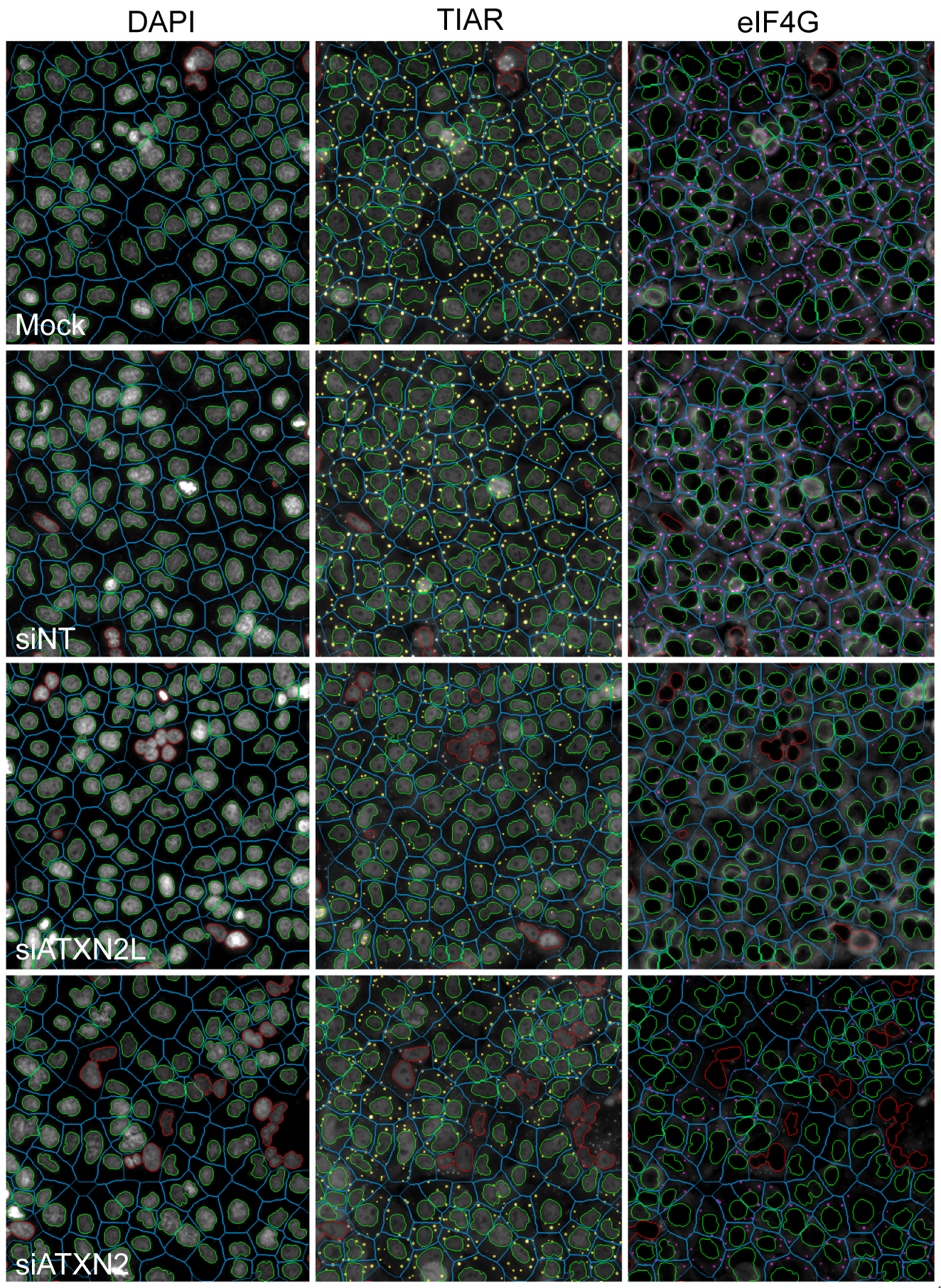

Supplement: Figure S4 — Quantification of TIAR- and eIF4G-positive SGs. HeLa cells were left untreated (mock) or transfected with non-targeting (siNT), or ATXN2L- or ATXN2-specific siRNA molecules. 72 h post transfection cells were treated with 0.5 mM sodium arsenite for 1 hour to induce SG formation, fixed, and stained with TIAR- and eIF4G-specific antibodies. Cell nuclei were stained with DAPI. Representative view fields of the automated image analysis are shown. Green encircled nuclei were selected; red encircled nuclei were rejected by the object identification algorithm. Outer cell borders (blue lines) were calculated by extending the nuclear region. TIAR-positive (yellow) and eIF4G-positive (magenta) SGs were quantified within whole cells. (TIF) [file pone.0050134.s004.tif]

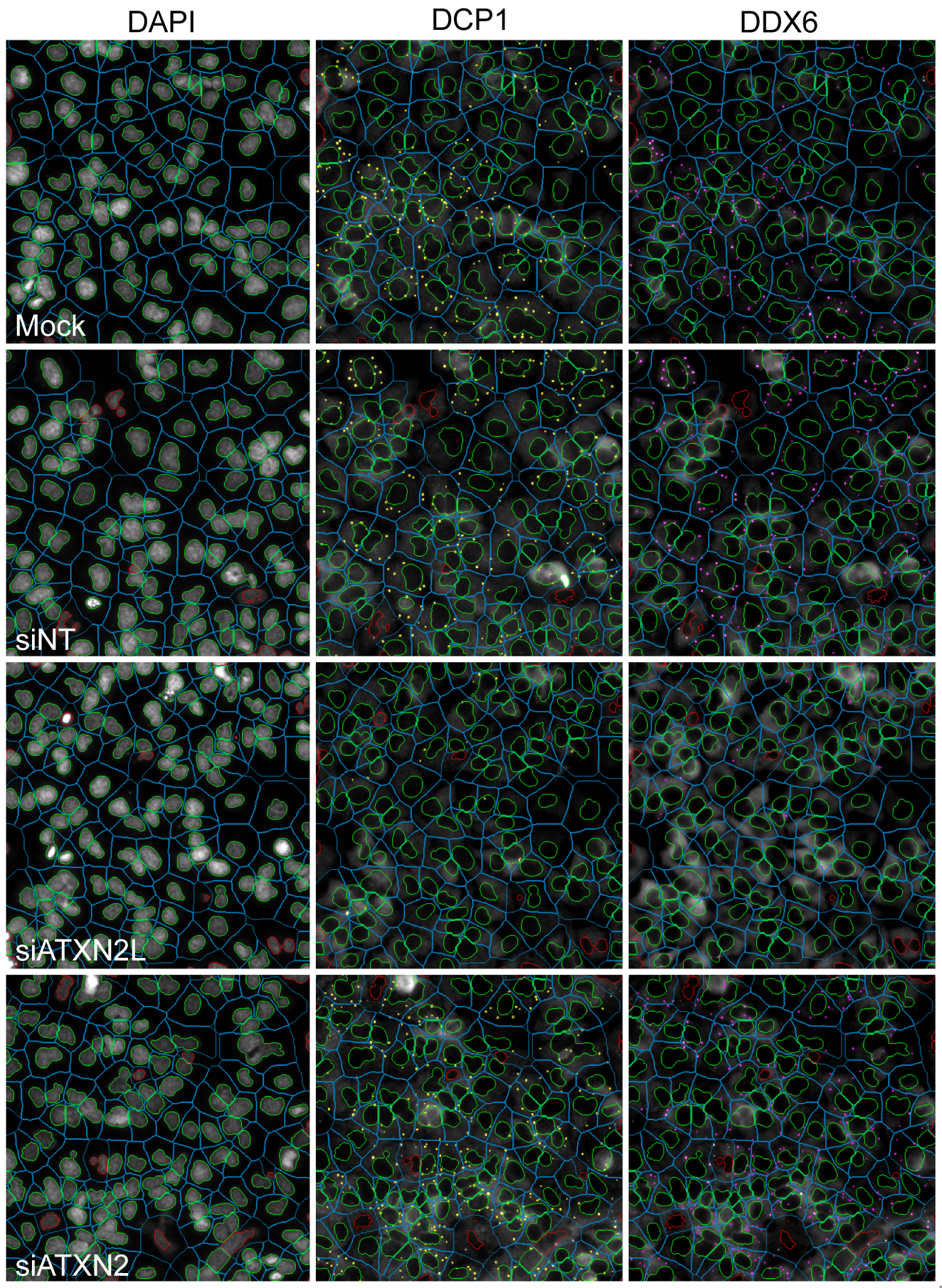

Supplement: Figure S5 — Quantification of DCP1- and DDX6-positive P-bodies. HeLa cells were left untreated (mock) or transfected with non-targeting (siNT), ATXN2L- or ATXN2-specific siRNA molecules. 72 h post transfection cells were fixed and stained with DCP1- and DDX6-specific antibodies. Cell nuclei were stained with DAPI. Representative view fields of the automated image analysis are shown. Green encircled nuclei were selected; red encircled nuclei were rejected by the object identification algorithm. Outer cell borders (blue lines) were calculated by extending the nuclear region. DCP1-positive (yellow) and DDX6-positive (magenta) P-bodies were quantified within whole cells. (TIF) [file pone.0050134.s005.tif]
